# Association between the expression status of programmed cell death ligand 1 and the efficacy of pan-cancer neoadjuvant immune checkpoint blockade

**DOI:** 10.3389/fimmu.2025.1617905

**Published:** 2025-09-30

**Authors:** Ying Huang, Junxing Xie, Jing Wang, Jingyi Lin, Meiling Chen, Bin Zhao, Zhiyang Huang

**Affiliations:** Quanzhou First Hospital Affiliated to Fujian Medical University, Quanzhou, China

**Keywords:** cancer, immunotherapy, PD-L1, neoadjuvant therapy, pathologic completeresponse, event-free survival

## Abstract

**Background:**

Immune checkpoint inhibitors (ICIs)-based neoadjuvant therapy has been regulatory approved in clinical practice since 2021. However, it is still difficult to determine which patients can benefit from it. Here, we conducted a meta-analysis to evaluate the predictive values of programmed cell death ligand 1 (PD-L1) in pan-cancer neoadjuvant immunotherapy.

**Methods:**

We searched MEDLINE and EMBASE for randomized controlled trials (RCTs) to collect information regarding pathological complete response (pCR) and event-free survival (EFS) in patients with PD-L1-positive and PD-L1-negative tumors. Odd ratio (OR), hazard ratio (HR), and their 95% confidence intervals (CIs) were calculated.

**Results:**

Totally, 10353 patients with 6 tumor types in 23 RCTs were included in this study. Neoadjuvant immunotherapy was associated with increased pCRs in both patients with PD-L1-positive (OR, 3.22; 95% CI, 2.25-4.61; *P* < 0.001) and PD-L1-negative tumors (OR, 2.07; 95% CI, 1.42-3.00; *P* < 0.001). However, compared with PD-L1 negative tumors, PD-L1 positive tumors benefited more from ICB-based neoadjuvant therapy (interaction effect, 0.65; 95% CI, 0.45-0.94; *P_Interaction_* = 0.01). Similarly, neoadjuvant immunotherapy resulted in favorable EFS in patients with PD-L1 positive (HR, 0.55; 95% CI, 0.46-0.66; *P* < 0.001) and PD-L1 negative tumors (HR, 0.70; 95% CI, 0.62-0.80; *P* < 0.001), the efficacy differences were also significant (interaction effect, 1.24; 95% CI, 1.03-1.50; *P_Interaction_* = 0.04).

**Conclusion:**

Both patients with PD-L1-positive and PD-L1-negative tumors can benefit from neoadjuvant immunotherapy. However, the magnitude of efficacy is greater in patients with PD-L1-positive tumors. Accordingly, rather than serving as an independent marker for patient selection, PD-L1 expression is more effectively applied as a prognostic biomarker.

## Introduction

Immune checkpoint inhibitors (ICIs) targeting programmed cell death protein 1 (PD-1), programmed cell death ligand 1 (PD-L1), and cytotoxic T-lymphocyte-associated antigen 4 (CTLA-4) can significantly prolong the overall survival (OS) and have been standard therapeutic agents in a number of malignancies (1). In recent years, immune checkpoint blockade (ICB)-based neoadjuvant therapy has been gradually recognized as a potential treatment approach for various tumors. Accumulating evidence revealed that neoadjuvant immunotherapy, by promoting systemic anti-cancer immunity, was associated with eliminating potential micro-metastases, reduction of the tumor stages, improvement of the R0 resection rate, and enhancement of pathological responses (2). This notion differs greatly from the well-established paradigm of conventional neoadjuvant therapy, which is simply known as a means to decrease tumor size. Currently, neoadjuvant immunotherapy has been regulatory granted in several malignancies, including breast cancer in 2021, lung cancer in 2022, and melanoma in 2023 (2). Meanwhile, hundreds of clinical trials have been launched involving various other indications, which will undoubtedly lead to more approvals in the future.

ICB-based neoadjuvant therapy may postpone surgery if the disease advances or potentially induce life-threatening immune-related toxicities (3). However, to date, it is still difficult to determine which patients should be offered neoadjuvant immunotherapy. Considering the fundamental nature of these agents, PD-L1 expression status is usually considered biologically plausible for predicting tumor response (4). Indeed, the European Medicines Agency grants the application of nivolumab-based neoadjuvant setting exclusively for patients with PD-L1 positive lung cancer (5). However, a considerable number of exceptions are recorded in clinical practice. For example, a recent study revealed that pathologic complete responses (pCRs) were reported in over 20% patients with PD-L1 negative breast cancer treated with ICB-based neoadjuvant therapy (3). Accordingly, the predictive values of PD-L1 expression status remain undetermined. To address this issue, here, we conducted a meta-analysis to systematically assess the efficacy of neoadjuvant immunotherapy in both patients with PD-L1-positive and PD-L1-negative tumors.

## Method

### Search strategy and selection criteria

A systematic search of MEDLINE and EMBASE databases for published trials on neoadjuvant ICB in patients with solid tumors from inception to March 2025 was conducted without language restriction. In addition, abstracts from the American Society of Clinical Oncology conference, European Society for Medical Oncology conference, and American Association for Cancer Research conference were examined for potential updates on published trials. The keywords used for search included: cancer, clinical trial, neoadjuvant, immunotherapy, PD-1, and PD-L1.

Both inclusion and exclusion standards were pre-specified. To be eligible, studies had to meet the following criteria: (1) study design: randomized controlled trials (RCTs) irrespective of clinical phase; (2) population: over 18 years of age, had histologic confirmation resectable solid tumors; (3) intervention: at least one experimental arm of patients who were treated with neoadjuvant immunotherapy (monotherapy or combination strategy) irrespective of dosage or duration, and one control arm with treatment did not involve any ICIs; (4) outcomes: pCR and event-free survival (EFS) in both patients with PD-L1 positive and PD-L1 negative tumors. Studies were excluded if they were: (1) other studies on this topic, including review articles, retrospective studies, editorials, letters, nonclinical or pre-clinical papers, phase I and non-randomized phase II studies, comments, quality of life studies, and cost effectiveness analyses; (2) studies in the pediatric population, patients with hematological disease, or small sample size (n<50); (3) patients with active central nervous system metastases, autoimmune disease, and glucocorticoid or immunosuppressant use; (4) studies with irretrievable or insufficient information for our statistical analysis.

All investigators independently conducted the initial search, reviewed the title and abstract for relevance, and classified the potential papers as included, uncertain, and excluded. For uncertain trials, the full texts were examined for the confirmation of eligibility. When multiple publications of the same trial appeared, only the most recent and/or complete report was included in our analysis.

### Data extraction and quality assessment

Relevant data were extracted independently by all investigators using a prespecified form. Extracted information were listed as follows: (1) study information, including study design, clinical phase, randomization stratified by PD-L1 expression status, PD-L1 detective method, neoadjuvant treatment regimens, and the intention-to-treat sample size; (2) baseline characteristics of the included patients, including age, cancer type, the definition of PD-L1 positive, and numbers of patients with PD-L1 positive and PD-L1 negative tumors, respectively; (3) data on treatment-related outcomes, including the number of patients who achieved pCR and information regarding EFS. Hazard ratios (HRs) for EFS and their 95% CIs stratified by PD-L1 level were extracted from each included study. Only studies that reported the outcome of interest were included in the relevant analysis.

Risk of bias was assessed by the Cochrane risk of bias tool (6). We examined every trial and scored it as high, low, or unclear risk of bias to the following criteria: random sequence generation; allocation concealment; blinding of participants and personnel to the study protocol; blinding of outcome assessment; incomplete outcome data; and selective reporting.

When disagreements occurred in terms of study selection, data extraction, and risk of bias assessment, all investigators double-checked the original data independently and discussed the potential problems together. The discrepancies were resolved when all authors came to an agreement.

### Statistical analysis

The primary endpoints were the improvements of pCR and EFS in patients with PD-L1-positive and PD-L1-negative tumors who were treated with neoadjuvant immunotherapy compared with conventional treatment. Statistical heterogeneity between different trials and subgroups was evaluated by Cochrane’s Q statistic (7). The *I* ^2^ statistic was estimated to evaluate the extent of inconsistency attributable to the heterogeneity across different trials. The assumption of homogeneity was considered invalid for *I* ^2^ > 50% and *P* < 0.10. The heterogeneity of efficacy between patients who were PD-L1 positive and PD-L1 negative was assessed by an interaction test and expressed as *P* for interaction. To explore the potential sources of heterogeneity and to examine the influence of different exclusion standards on the overall efficacy of neoadjuvant immunotherapy, pre-defined subgroup analyses were also performed. In this study, the sensitivity analysis was conducted according to different masking methods, cancer type, drug target, randomization stratified by PD-L1 expression status, and clinical phase. Publication bias was evaluated through visual inspection of Begg’s funnel plots (8). The Egger linear regression test and the Begg rank correlation test were further conducted with the significance of *P* < 0.10 (8, 9).

All analysis was conducted by Stata version 12.0 and MedCalc 18.2.1 software. Two-sided *P* < 0.05 was considered statistically significant.

## Results

### Baseline characteristics of the included trials

1514 relevant manuscripts were discovered from the initial search, including 879 studies from MEDLINE and 635 articles from EMBASE. Further examinations removed 1491 papers that failed to meet our inclusion criteria (**Supplementary Figure S1**). Totally, 10353 patients with 6 tumor types in 23 RCTs were included in this study (**Table 1**), with 4976 (48.1%) patients as controls, and 5377 patients (51.9%) in the experimental arms. Patients received agents targeting PD-1 in 14 trials (including camrelizumab in 5 RCTs, nivolumab in 3 trials, pembrolizumab in 3 studies, and sintilimab, toripalimab, and tislelizumab in 1 RCT each), agents targeting PD-L1 in 8 trials (including atezolizumab in 5 studies and durvalumab in 3 RCTs), and the combination of nivolumab and ipilimumab in 1 RCT. Among 23 eligible trials, 18 studies with 9816 patients (94.8%) were phase 3 RCTs, the rest 5 trials with 537 subjects (5.2%) were phase 2 studies. Breast cancer (n=4768) was investigated in 10 studies, lung cancer (n=3434) in 7 RCTs, gastric cancer (n=464) and rectal cancer (n=365) in 2 trials, and bladder cancer (n=1063) and esophageal cancer (n=259) in 1 RCT. According to the trial designs, the randomization stratified by PD-L1 expression status was conducted in 14 trials with 7792 cancer patients (75.3%). Totally, 6251 patients with PD-L1-positive tumors and 3750 subjects with PD-L1-negative tumors were identified. Among them, 3275 patients with PD-L1-positive cancer and 1904 subjects with PD-L1-negative cancer were treated with neoadjuvant ICB, while 2976 patients with PD-L1-positive diseases and 1846 subjects with PD-L1-negative diseases were in the control arms.

**Table 1:** Baseline characteristics of eligible randomized trials.

Study	Masking	Phase	Cancer type	Randomization stratified by PD-L1	PD-L1 antibody clone	Cut-off value	Treatment	No. of patients	Median age (range, year)	No. of PD-L1+/PD-L1-
ABCSG-52 (41)	Open-label	2	Breast cancer	No	SP124	IC=1%	Atezolizumab+targeted therapy	29	57(33-77)	12/15
							Targeted therapy	29	58(38-82)	17/11
AEGEAN (42)	Double-blind	3	Lung cancer	Yes	SP263	TC=1%	Durvalumab+chemotherapy	366	65(30-88)	244/122
							Chemotherapy	374	65(39-85)	249/125
Arise-FJ-G005 (43)	Open-label	2	Gastric cancer	No	SP263	CPS=1	Camrelizumab+chemotherapy	51	63(57-68)	27/22
							Chemotherapy	53	63(56-68)	27/23
CamRelief (44)	Double-blind	3	Breast cancer	Yes	E1L3N	CPS=10	Camrelizumab+chemotherapy	222	49(22-72)	127/95
							Chemotherapy	219	48(22-75)	127/92
CheckMate 7FL (45)	Double-blind	3	Breast cancer	Yes	SP142	IC=1%	Nivolumab+chemotherapy	257	50(24-78)	88/169
							Chemotherapy	252	51(23-79)	84/169
CheckMate 77T (46)	Double-blind	3	Lung cancer	Yes	28–8 pharmDx	TC=1%	Nivolumab+chemotherapy	229	66(37-83)	128/93
							Chemotherapy	232	66(35-86)	128/93
CheckMate 816 (47, 48)	Open-label	3	Lung cancer	Yes	28–8 pharmDx	TC=1%	Nivolumab+chemotherapy	179	64(41-82)	89/78
							Chemotherapy	179	65(34-84)	89/77
CheckMate 816-2 (49)	Open-label	3	Lung cancer	Yes	28–8 pharmDx	TC=1%	Nivolumab+ipilimumab	113	64(34-83)	60/49
							Chemotherapy	108	65(34-86)	58/43
DRAGON IV (50)	Open-label	3	Gastric cancer	No	NA	CPS=1	Camrelizumab +chemotherapy	180	63(28-75)	85/58
							Chemotherapy	180	63(34-75)	92/50
ESCORT-NEO (51)	Open-label	3	Esophageal cancer	No	22C3 pharmDx	CPS=10	Camrelizumab+chemotherapy	130	63(44-75)	40/39
							Chemotherapy	129	65(44-75)	80/72
GeparNuevo (52, 53)	Double-blind	2	Breast cancer	No	SP263	TC/IC=1%	Durvalumab+chemotherapy	88	50(25-74)	69/9
							Chemotherapy	86	50(23-76)	69/11
IMpassion031 (54, 55)	Double-blind	3	Breast cancer	Yes	SP142	IC=1%	Atezolizumab+chemotherapy	165	51 (22–76)	78/87
							Chemotherapy	168	51 (26–78)	76/92
IMpassion050 (56, 57)	Double-blind	3	Breast cancer	Yes	SP142	IC=1%	Atezolizumab+ddAC-PacPH	228	50	109/119
							Placebo+ddAC-PacPH	226	50	110/116
KEYNOTE-522 (58–60)	Double-blind	3	Breast cancer	No	22C3 pharmDx	CPS=1	Pembrolizumab+chemotherapy	784	49 (22-80)	656/128
							Chemotherapy	390	48 (24-79)	317/69
KEYNOTE-671 (35, 36)	Double-blind	3	Lung cancer	Yes	22C3 pharmDx	TPS=50	Pembrolizumab+chemotherapy	397	63(26-83)	132/265
							Chemotherapy	400	64(35-81)	134/266
KEYNOTE-756 (61)	Double-blind	3	Breast cancer	Yes	22C3 pharmDx	CPS=1	Pembrolizumab+chemotherapy	635	49(24-82)	482/153
							Chemotherapy	643	49(19-78)	489/154
NCI 10013 (62)	Open-label	2	Breast cancer	No	SP142	IC=1%	Atezolizumab+chemotherapy	45	54	16/19
							Chemotherapy	22	49	4/5
NCT04304209 (63)	Open-label	2	Rectal cancer	No	22C3 pharmDx	CPS=2	Sintilimab+chemoradiotherapy	67	56(33-73)	39/25
							Chemoradiotherapy	67	56(25-72)	38/15
Neotorch (64)	Double-blind	3	Lung cancer	Yes	JS311	TC=1%	Toripalimab+chemotherapy	202	62(56-65)	133/51
							Chemotherapy	202	61(56-65)	132/54
NeoTRIP (65, 66)	Open-label	3	Breast cancer	Yes	SP142	IC=1%	Atezolizumab+chemotherapy	138	50(25-79)	79/59
							Chemotherapy	142	50 (24-77)	77/65
NIAGARA (67)	Open-label	3	Bladder cancer	Yes	SP263	TC/IC=25%	Durvalumab+chemotherapy	533	65(34-84)	389/144
							Chemotherapy	530	66(32-83)	388/142
RATIONALE-315 (68)	Double-blind	3	Lung cancer	Yes	SP263	TC=1%	Tislelizumab+chemotherapy	226	62(57-67)	130/89
							Chemotherapy	227	63(56-68)	132/84
UNION (69)	Open-label	3	Rectal cancer	No	22C3 pharmDx	CPS=1	Camrelizumab+ chemotherapy	113	58(32-75)	63/16
							Chemotherapy	118	58(31-75)	59/18

CPS, combined positive score; IC: immune cell; TC: tumor cell; TPS, tumor proportion score; NA, not available.

The method qualities of the eligible RCTs, assessed by the Cochrane risk of bias tool (6), were generally moderate to good; the major issue was lack of blinding since 11 trials were open-labelled.

### Efficacy of neoadjuvant immunotherapy and PD-L1 expression status

**Figure 1:**
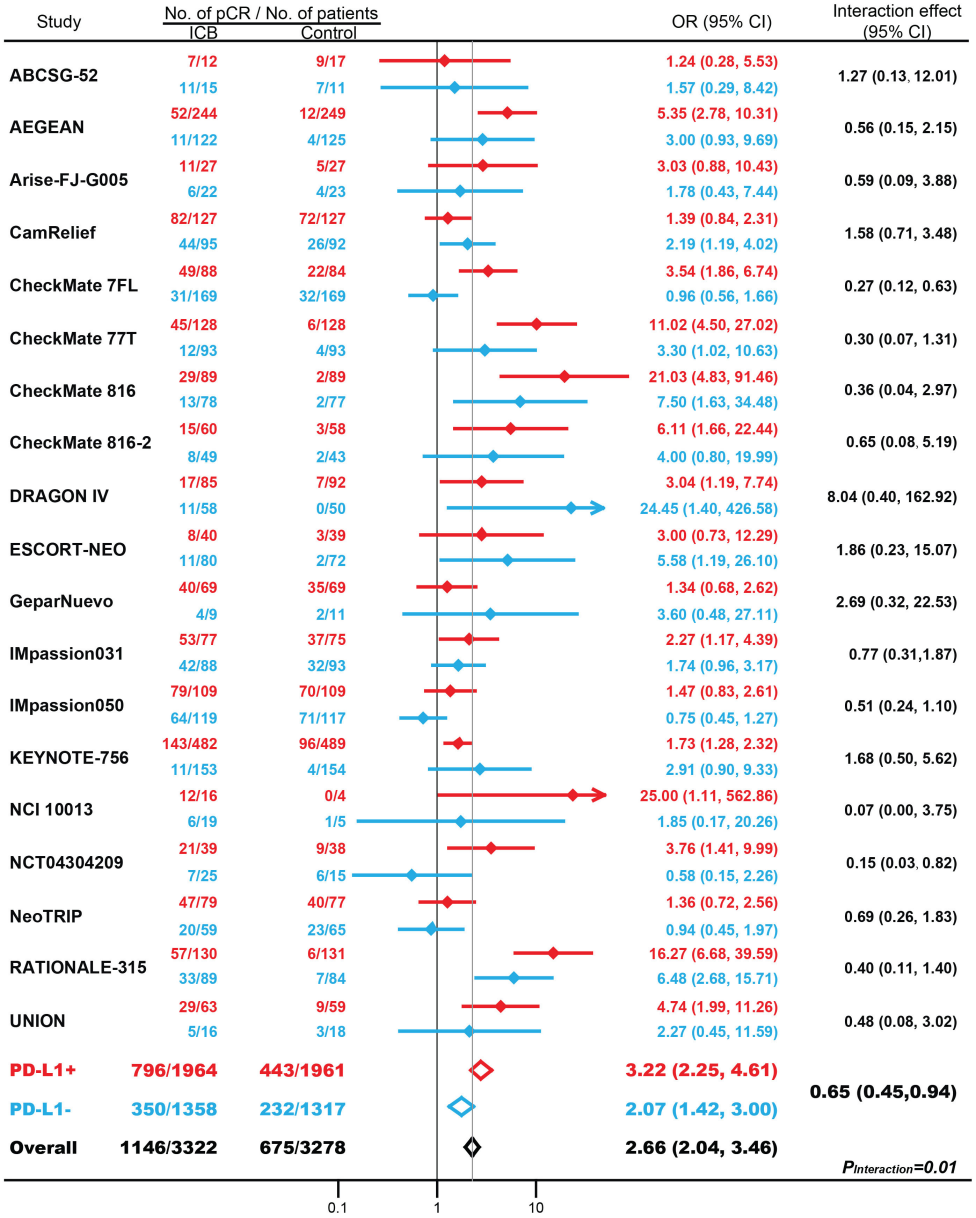
The association between PD-L1 expression status and pathological complete response (pCR) in patients treated with immune checkpoint blockade (ICB)-based neoadjuvant therapy. OR, odds ratio. Red indicates patients with PD-L1-positive tumors; Blue indicates patients with PD-L1-negative tumors.

Overall, in 19 RCTs with 6600 patients, compared with conventional treatment, ICB-based neoadjuvant therapy was associated with more pCRs (34.5% vs. 20.6%; odds ratio [OR], 2.66; 95% CI, 2.04-3.46; *P* < 0.001; **Figure 1**). There were significantly increased pCRs in both patients with PD-L1-positive (40.5% vs. 22.6%; OR, 3.22; 95% CI, 2.25-4.61; *P* < 0.001) and patients with PD-L1-negative tumors (25.8% vs. 17.6%; OR, 2.07; 95% CI, 1.42-3.00; *P* < 0.001). Of note, the magnitude of efficacies was greater in patients with PD-L1 positive tumors compared with patients with PD-L1 negative tumors (interaction effect, 0.65; 95% CI, 0.45-0.94; *P_Interaction_* = 0.01). Subgroup analysis based on masking method, clinical phase, cancer type, drug target, and randomization stratified by PD-L1 status, showed similar results but to a lesser extent (**Supplementary Figure S2**).

Totally, 16 eligible RCT examined the association between PD-L1 expression and pCR with the thershold for PD-L1 expression was set as 1%. 1758 patients with PD-L1 positive tumors and 1158 individuals with PD-L1 negative tumors were treated with immune checkpoint inhibitors, while 1757 patients with PD-L1 positive tumors and 1138 individuals with PD-L1 negative tumors were included in the control arms. For patients with PD-L1 positive tumors, 685 patients (39.0%) responded to ICB, while 359 pCRs (20.4%) were observed in control arms. The difference was significant (OR, 2.54; 95% CI, 2.14-3.02; *P* < 0.001). similarly, for PD-L1 negative tumors, more pCRs were identified in patients treated with ICB (n=288, 24.9%) than in control arms (n=198, 17.4%) (OR, 1.57; 95% CI, 1.24-1.99; *P* = 0.002). The magnitude of efficacies was greater in patients with PD-L1 positive tumors compared with patients with PD-L1 negative tumors (*P_Interaction_* = 0.001).

**Figure 2:**
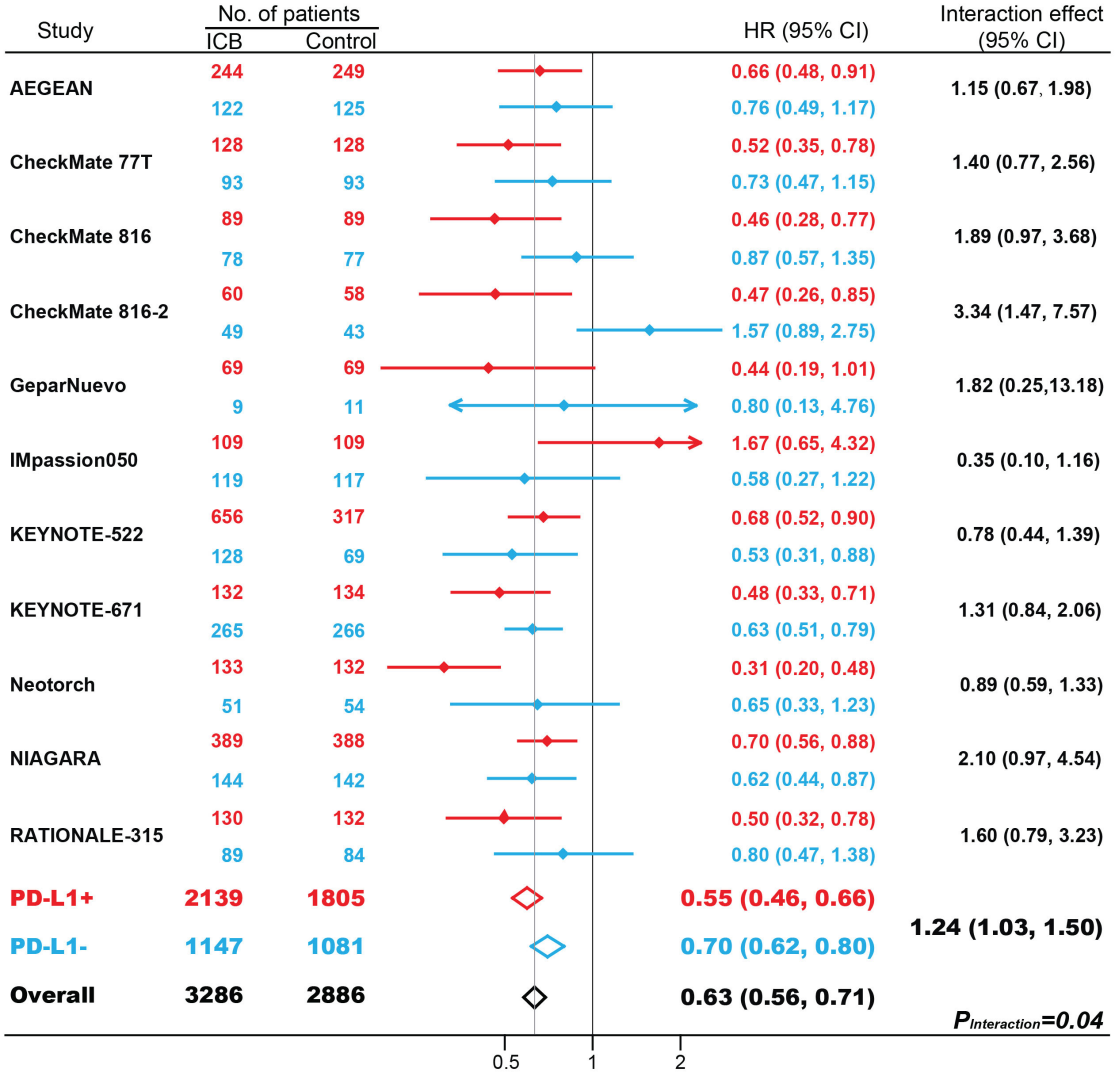
The impact of PD-L1 expression on event-free survival (EFS) in patients treated with immune checkpoint blockade (ICB)-based neoadjuvant therapy. HR, hazard ratio. Red indicates patients with PD-L1-positive tumors; Blue indicates patients with PD-L1-negative tumors.

In 11 studies with 6172 patients, ICB-based neoadjuvant therapy was associated with favorable EFS (hazard ratio [HR], 0.63; 95% CI, 0.56-0.71; *P* < 0.001; **Figure 2**). The efficacies of neoadjuvant immunotherapy were significantly improved in both patients with PD-L1-positive (HR, 0.55; 95% CI, 0.46-0.66; *P* < 0.001) and PD-L1-negative tumors (HR, 0.70; 95% CI, 0.62-0.80; *P* < 0.001). Patients with PD-L1-positive tumors benefit more from neoadjuvant immunotherapy compared with patients with PD-L1-negative tumors (interaction effect, 1.24; 95% CI, 1.03-1.50; *P_Interaction_* = 0.04). Further subgroup analysis based on masking method, cancer type, drug target, and randomization stratified by PD-L1 status, was shown in **Supplementary Figure S3**. Interestingly, partly due to fewer trials included, the superiority of EFS benefits in PD-L1-positive tumors over PD-L1-negative tumors was not as great as the pCR benefits.

No significant asymmetry was identified by visual inspection of Begg’s funnel plot (**Supplementary Figure S4**).

## Discussion

For the first time to our knowledge, this study, based on high-quality RCTs including the largest sample size to date, reveals that both patients with PD-L1-positive and PD-L1-negative tumors can benefit from neoadjuvant immunotherapy. However, it should be noted that, compared with patients with PD-L1-negative tumors, the magnitudes of efficacy are greater in patients with PD-L1-positive tumors. Considering hundreds of neoadjuvant ICB trials are currently underway, these findings may serve as a valuable reference in the drug development process, aid in the design and interpretation of clinical trials, and provide complementary information in drafting the clinical practice guideline.

Previous investigations have validated the importance of PD-L1 expression in forecasting the effectiveness of ICIs in advanced patients, showing a positive correlation between PD-L1 levels and the benefits of immunotherapy (10). The absence of PD-L1 expression is commonly assumed to result in weak or no anti-tumor immunity induced by ICIs. In our previous study (4), we investigated 4174 patients enrolled in 8 randomized trials; 2254 had PD-L1-positive tumors, and the other 1920 individuals were PD-L1-negative. All patients had advanced or metastatic diseases, and they were diagnosed as lung cancer, melanoma, renal cell carcinoma, head and neck cancer, and urothelial carcinoma. Inhibitors targeting PD-1/PD-L1 were administered as second-line or later treatment in these subjects. Our results revealed that, compared with conventional treatment, immunotherapy was associated with favorable overall survival in patients with PD-L1-negative tumors (HR, 0.80; 95% CI, 0.71-0.90; P<0.001). Moreover, one recent study conducted in 5569 patients with lung cancer across different PD-L1 levels suggested that the improved efficacy of immunotherapy was independent of PD-L1 expression status in neoadjuvant, adjuvant, and peri-operative settings (11). Similarly, in the present study, our analysis revealed that, for subjects lacking PD-L1 expression, ICB-based neoadjuvant therapy was associated with better outcomes compared with conventional treatment. This result, based on well-defined endpoints of pCR and EFS from 3750 patients who did not express PD-L1 in high-quality RCTs, enhanced our analysis by mitigating the problem of individual trials lacking sufficient power. Technical explanations may account for why patients with negative PD-L1 expression can also gain advantages: the PD-L1 condition at treatment time may not be accurately depicted by testing archived tissues after cancer progression; the availability of tissues to examine PD-L1 expression is restricted; or there may be inconsistencies in PD-L1 expression among different tumor histologies.

It is well-established that chemotherapy, radiotherapy, or even immunotherapy itself can foster the upregulation of PD-L1 expression, induce immunogenicity by improving antigen processing machinery and T-cell killing in tumor tissues (12, 13). The reason why more patients benefit from ICB-based neoadjuvant therapy may be that ICIs are combined with other agents. Indeed, certain chemotherapy drugs, like Oxaliplatin, are capable of causing immunogenic cell death (ICD) and suppressing tumor growth by enhancing T cell infiltration and activating dendritic cells within the tumor (14). Further *in vivo* studies demonstrated that the combination of Oxaliplatin and immune checkpoint inhibitors could improve the therapeutic outcomes (15). Interestingly, this synergistic effect of oxaliplatin-based chemotherapy and immunotherapy was not observed in the combination of cisplatin-based chemotherapy and immunotherapy in patients with gastric cancer (16). Indeed, accumulating evidence has demonstrated that various chemotherapy agents can induce immunogenic cell death (ICD) in tumors, such as Anthracyclines (doxorubicin and mitoxantrone) (17, 18), DNA-damaging agents (cyclophosphamide, platinum derivatives) (19–23), proteasome inhibitors (bortezomib) (24, 25), and paclitaxel (26). Other conventional treatments, including therapeutic oncolytic virus, targeted anti-tumor drugs, radiotherapy, external phototherapy, and photodynamic therapy, may also produce ICD (27). The immunogenic microenvironment created by drug-induced ICD can significantly enhance the efficacy of ICB. Additionally, the HMGB1 released during the ICD process may trigger the rapid endocytosis and subsequent breakdown in lysosomes, which in turn improves T cell anti-cancer activities, though inhibiting the persistent signal transduction of PD-1 (28). Apart from directly inhibiting the growth of cancer cells, chemoradiotherapy can also activate immune effectors, boosting anti-cancer immune response while the bulk tumor and tumor antigens still remain during treatment (29). Furthermore, the neoantigens resulting from neoadjuvant therapy will provoke a vigorous and enduring anti-cancer reaction, even following surgical procedures (30). Moreover, PD-L2 serves as another important ligand for PD-1, inhibiting the function of T cells and contributing to tumor immune escape, binding to PD-1 with 2–6 times greater affinity than PD-L1 (31). However, the prognostic or predictive significance of PD-L2 in cancer has yet to be determined. Therefore, future studies should prioritize promoting the whole tumor immune microenvironment, rather than solely concentrating on PD-L1 expression.

The molecular mechanisms underlying PD-L1 expression and chemotherapy are complex and still largely unclear. For example, approximately half of the patients with non-small-cell lung cancer have negative PD-L1 expression (32), but numerous studies have suggested that individuals in this subpopulation can benefit significantly from immunotherapy compared with conventional chemotherapy. This may be attributed to the unique molecular PD-L1 signaling pathways or clinical features associated with chemotherapy rather than the tumor immune microenvironment itself. Previous studies reported that Mutations in *STK11* and *EGFR*, as well as alterations in the WNT pathway, have been strongly linked to negative PD-L1 expression, whereas mutations in *TP53*, *KRAS*, and *MET* have been robustly associated with high PD-L1 expression (33). Moreover, low PD-L1 expression levels are correlated with particular clinicopathological characteristics such as primary tumors, adenocarcinoma, and resected samples (34).

There are several issues regarding ICB-based neoadjuvant therapy that still need to be addressed. First, neoadjuvant immunotherapy alone and the combination of neoadjuvant and adjuvant immunotherapy are the two major treatment managements based on the eligible RCTs. Nevertheless, the selection of the most effective treatment for cancer patients is still debated. Currently, there is a deficiency of studies on whether continuing immunotherapy post-surgery prolongs survival compared to pre-operative use alone, and the best duration for post-operative immunotherapy remains unclear. It is important to identify which patients should continue immunotherapy post-surgery in future studies, and novel biomarker analysis, like circulating DNA, may be needed. Second, there is ambiguity about the requirement of chemotherapy for patients with PD-L1 expression levels of 50% or higher in the neoadjuvant settings. In KEYNOTE-671 (35, 36), the cut-off value for PD-L1 expression status was set as 50%. Immunotherapy was associated with favorable EFS in both patients with PD-L1-negative tumors (HR, 0.64; 95% CI, 0.49-0.82) and patients with PD-L1-positive tumors (HR, 0.42; 95% CI, 0.28-0.65). The findings were in agreement with our major conclusion. Unfortunately, all RCTs have been set up to make a direct comparison between ICB-based immunotherapy and conventional treatment. Of note, besides evaluating the synergistic interaction between chemotherapy and immunotherapy (37), the potential toxicities associated with chemotherapy should also be taken into account. Further clinical studies are necessary to address these concerns.

Our study also has some limitations. First, it is essential to assess whether neoadjuvant immunotherapy can convert the pCR/EFS advantage into a substantial overall survival improvement over conventional treatment, as it defines the ultimate purpose of neoadjuvant immunotherapy. However, since the information regarding overall survival in these eligible RCTs is immature, we cannot conduct such an analysis here. Second, the detection methods of PD-L1 expression status were conducted by various approaches. However, numerous studies have investigated the reproducibility of PD-L1 interpretation concordance among different tumor types. For example, the Blueprint Project phase 2 has revealed a high concordance among staining tests of Dako 22C3, Ventana SP263, and Dako 28–8 in lung cancer (38), Hodgson et al. demonstrated good analytic comparability of Ventana SP142, Ventana SP263, Dako 22C3, and CST E1L3N in urothelial carcinoma (39) and esophageal cancer (40). Hence, we believe the potential bias due to different PD-L1 antibodies was acceptable here. Third, other confounding factors, such as tumor types, drug targets, and imbalances in patients’ characteristics among PD-L1-positive and PD-L1-negative tumors, may also be the source of heterogeneity. Accordingly, we conducted pre-defined subgroup analysis based on the classifications of these features, and found no significant differences in terms of pCR and EFS among these subgroups. Fourth, the treatment regimens, patterns, cycles, and duration varied greatly in the eligible trials, which may introduce some selection bias. Moreover, our analysis was conducted at the trial level. Accordingly, individualized patient data were urgently needed to confirm our results. Fifth, although patients with PD-L1-negative tumors can benefit from immunotherapy, determining the optimal management strategy for cancer patients necessitates a multifaceted process in real-world clinical practice. The toxicity profile and financial burdens were also key factors in choosing treatment options. However, it was very difficult to address these concerns due to the limited information. Hence, the clinicians need to carefully balance efficacy, safety, and patient preferences to deliver individualized treatment.

In summary, our meta-analysis demonstrates that both patients with PD-L1-positive and PD-L1-negative tumors can benefit from neoadjuvant immunotherapy. However, compared with patients with PD-L1-negative tumors, the magnitude of efficacy is greater in patients with PD-L1-positive tumors. Hence, rather than serving as an independent marker for patient selection, PD-L1 expression status is more effectively applied as a prognostic biomarker.

## Data Availability

The original contributions presented in the study are included in the article/**Supplementary Material**. Further inquiries can be directed to the corresponding author.

## Supplementary material

Supplementary Figure 1 Flowchart diagram of selected clinical trials included in our study.

Supplementary Figure 2 Subgroup analysis of the association between PD-L1 and pCR in patients treated with neoadjuvant immunotherapy. OR, odds ratio. Red indicates patients with PD-L1-positive tumors; Blue indicates patients with PD-L1-negative tumors.

Supplementary Figure 3 Subgroup analysis of the association between PD-L1 and EFS in patients treated with neoadjuvant immunotherapy. HR, hazard ratio. Red indicates patients with PD-L1-positive tumors; Blue indicates patients with PD-L1-negative tumors.

Supplementary Figure 4 Begg’s funnel plot for the publication bias test.
