# Supplementary figures and images for: Association between the expression status of programmed cell death ligand 1 and the efficacy of pan-cancer neoadjuvant immune checkpoint blockade
Source: Front Immunol. 2025 Sep 30;16:1617905. doi: 10.3389/fimmu.2025.1617905 (PMC12518071; doi:10.3389/fimmu.2025.1617905)

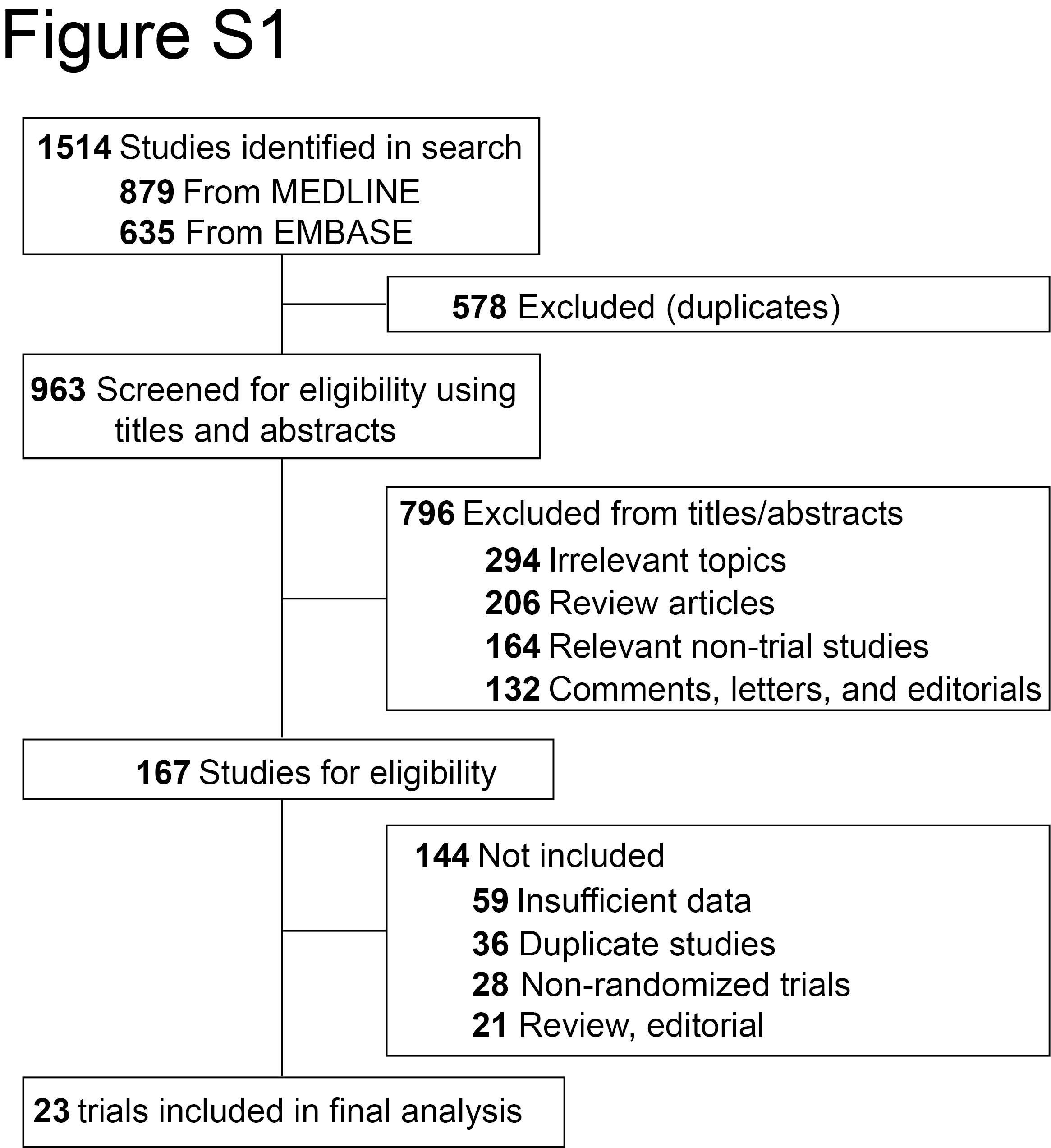

Supplement: Supplementary Figure 1 — Flowchart diagram of selected clinical trials included in our study. [file Image1.jpeg]

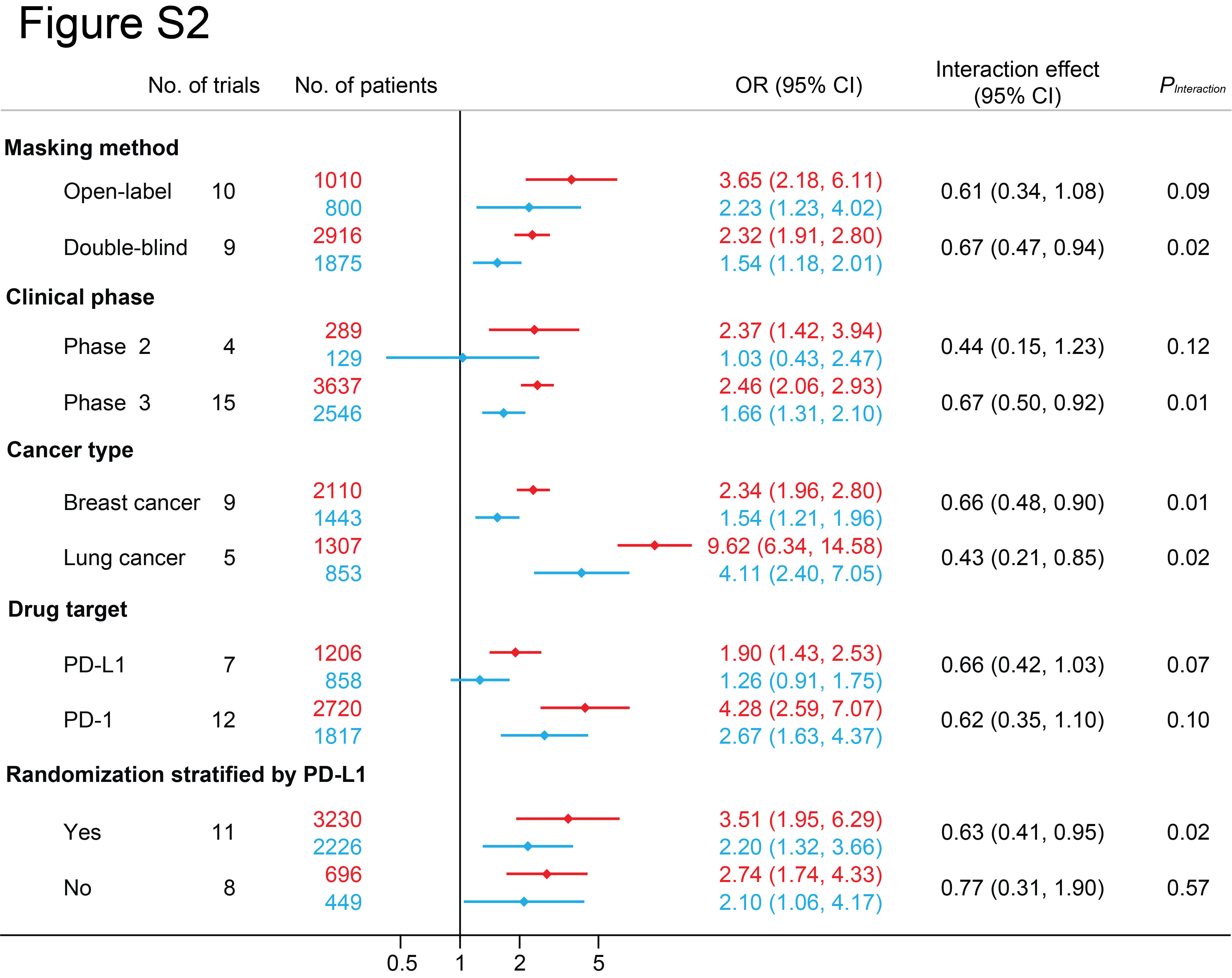

Supplement: Supplementary Figure 2 — Subgroup analysis of the association between PD-L1 and pCR in patients treated with neoadjuvant immunotherapy. OR, odds ratio. Red indicates patients with PD-L1-positive tumors; Blue indicates patients with PD-L1-negative tumors. [file Image2.jpeg]

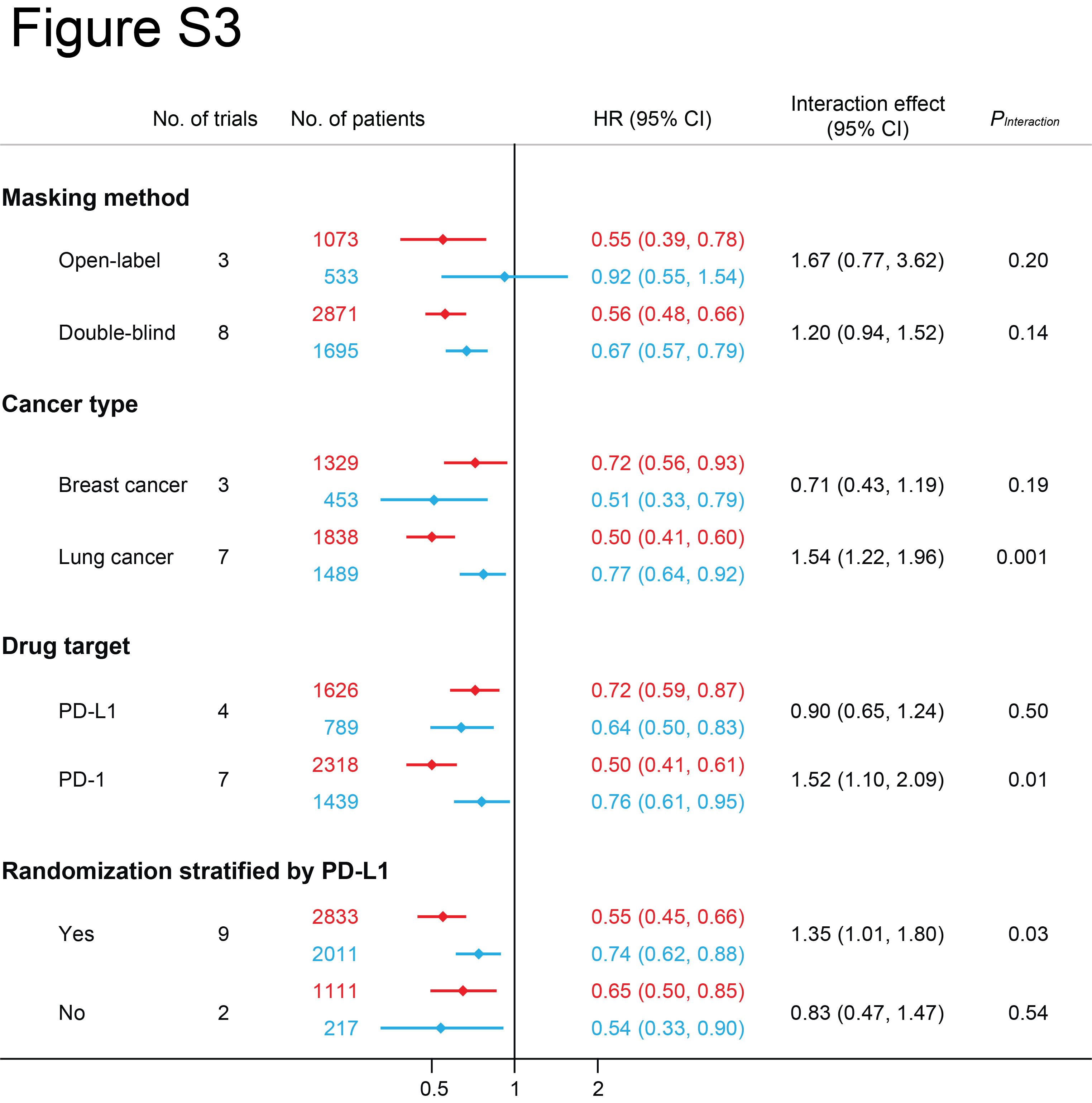

Supplement: Supplementary Figure 3 — Subgroup analysis of the association between PD-L1 and EFS in patients treated with neoadjuvant immunotherapy. HR, hazard ratio. Red indicates patients with PD-L1-positive tumors; Blue indicates patients with PD-L1-negative tumors. [file Image3.jpeg]

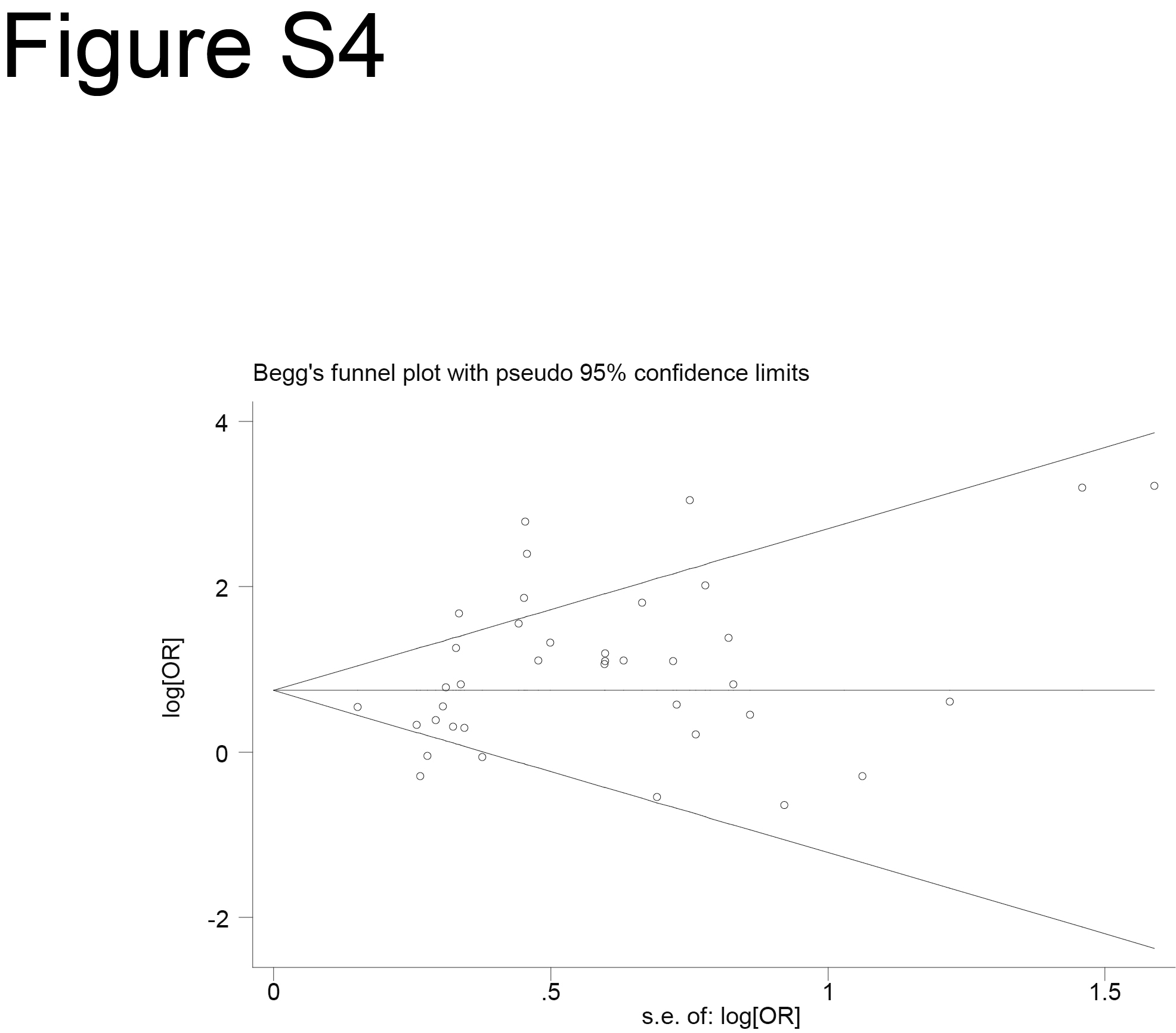

Supplement: Supplementary Figure 4 — Begg’s funnel plot for the publication bias test. [file Image4.jpeg]
